# First Description of Acaricide Resistance in Populations of *Rhipicephalus microplus* Tick from the Lower Amazon, Brazil

**DOI:** 10.3390/ani12212931

**Published:** 2022-10-26

**Authors:** Ana Beatriz Barbosa de Sousa, Daniela Bianchi, Elisa Mota Santos, Salatiel Ribeiro Dias, Poliana Leão Peleja, Raidel Reis Santos, Nohora Mercado Caruso, Antonio Humberto Hamad Minervino

**Affiliations:** 1 Laboratory of Animal Health, LARSANA, Federal University of Western Pará, UFOPA, Santarém 68040-255, Brazil; 2 Departamento Productividad e Innovación, Universidad de la Costa, CUC, Calle 58 n.55-66, Barranquilla 080001, Colombia

**Keywords:** acaricidal, efficiency, adult immersion test, susceptibility

## Abstract

**Simple Summary:**

Ticks represent a major health and economical issue for cattle farmers since bovine are infested by the cattle tick *Rhipicephalus microplui;* This tick is responsible for direct economic loss and can be the vector of several diseases. Many acaricidal drugs are routinely used to control tick infestation, but tick populations are becoming resistant to different drugs. Our work evaluated for the first time the resistance of a tick population from the Lower Amazon region to three different and commonly used acaricidal drugs. We found that the tested tick population had a marked resistance to Cypermethrin and Deltamethrin and mild resistance to Amitraz. Farmers in the region should avoid the use of such acaricides to control ticks, especially Cypermethrin and Deltamethrin.

**Abstract:**

There is limited information on the resistance to acaricidal drugs of the cattle tick Rhipicephalus microplus in the Lower Amazon region. Thus, we aimed to determine the efficiency of three widely used acaricide products (Amitraz, Cypermethrin, and Deltamethrin) in the control of this tick species. The adult immersion test was used on engorged female ticks sampled on farms in the Lower Amazon region, Brazil. For the test, homogeneous batches of 10 engorged females were placed in Petri dishes and immersed in the tested acaricidal drugs, using four replicates of each acaricide and three replicates as a negative control, immersed in distilled water. The acaricides were diluted as recommended by the manufacturers, and the ticks were submerged for five minutes. Mortality of engorged females, production of eggs, and percentage of larval hatching were evaluated. Tick mortality was 2.5%, 7.5%, and 0% for Amitraz, Cypermethrin, and Deltamethrin, respectively. The percentage of larval hatching was 53.7% for Amitraz, 88.7% for Cypermethrin, and 80.0% for Deltamethrin. As recommended by the FAO, for the acaricide to be considered effective, it must have a control rate ≥95%. Among the tested acaricides, Amitraz showed an efficacy of 90.5%, Cypermethrin 10.4%, and Deltamethrin 26.6%. Ticks from the lower Amazon showed marked resistance to Cypermethrin and Deltamethrin and mild resistance to Amitraz. This is the first report of acaricidal resistance in the region.

## 1. Introduction

*Rhipicephalus microplus*, known as the cattle tick, is one of the main parasites that affect cattle. It is estimated that 80% of the world’s livestock are infested by this tick [1], resulting in about US$ 3.24 billion of annual losses only in Brazil [2]. Tick infestation severely impacts productivity (e.g., reduced weight gain and milk production), increases farming costs due to treatments and leather devaluation, and can lead to animal losses [3]. In a recent study in the Brazilian Cerrado biome, simulated data showed an individual weight loss of more than 92 kg in cattle infested with ticks [4]. In addition, *R. microplus* is a vector of important diseases such as babesiosis and anaplasmosis [5].

The control of infestation by *R. microplus* depends on the interactions of several factors, such as environmental conditions, time of the year, management, and the breed of the host [6]. Chemical acaricides are the most used control method. In Brazil, the six most used classes of acaricidal drugs are organophosphates, synthetic pyrethroids, formamidines (Amitraz), macrocyclic lactones, phenylpyrazoles (fipronil), and benzoylphenylureas [7]. The frequent and inappropriate use of these products has contributed to the selection of resistant ticks, with a population of *R. microplus* resistant to all six acaricidal already found in Brazil [8].

A survey carried out with farmers in the western region of Pará showed little knowledge about the use of acaricides and the different acaricide chemical compounds available [9]. Organophosphates were one of the first acaricidal compounds reported to have resistant ticks [8]. Synthetic pyrethroids are widely used by farmers due to their prolonged residual activity and low toxicity for animals and humans [10], and due to their intense use, the resistance of parasites to this class has been observed in Brazil [11,12]. Amitraz, which emerged as a substitute for organophosphates and pyrethroids, has also shown a decrease in efficiency, as well as the macrocyclic lactones [13]. In 2014 [7] reported the first record of a population os *R. microplus* resistant to all acaricide classes in Brazil.

The northern region of Brazil is the second region with the largest cattle herd and has the largest buffalo herd in Brazil [14]. However, reports of acaricidal drug resistance are scarce. Only a few studies [15,16] in Rondônia, one state in the North Region, indicate the presence of *R. microplus* resistant to chemical acaricides. Pará is the most important state in terms of cattle farming, as it ranks third in the national herd, with 22.3 million heads [14]. To the best of our knowledge, there is no report of *R. microplus* tick population resistance to any acaricidal drug in the entire state of Pará.

The Lower Amazon region has a particular farming system with characteristic collective use of farming areas by cattle from different farms and owners in the wetlands with seasonal and regular cattle transportation [9]. This specific feature can contribute to disseminating the resistant genes amongst different tick populations in the region. Therefore, this study aimed to investigate the presence of strains of *R. microplus* resistant to the most commonly used commercial acaricides in the Lower Amazon.

## 2. Materials and Methods

### 2.1. Tick Collection Location

**Figure 1:**
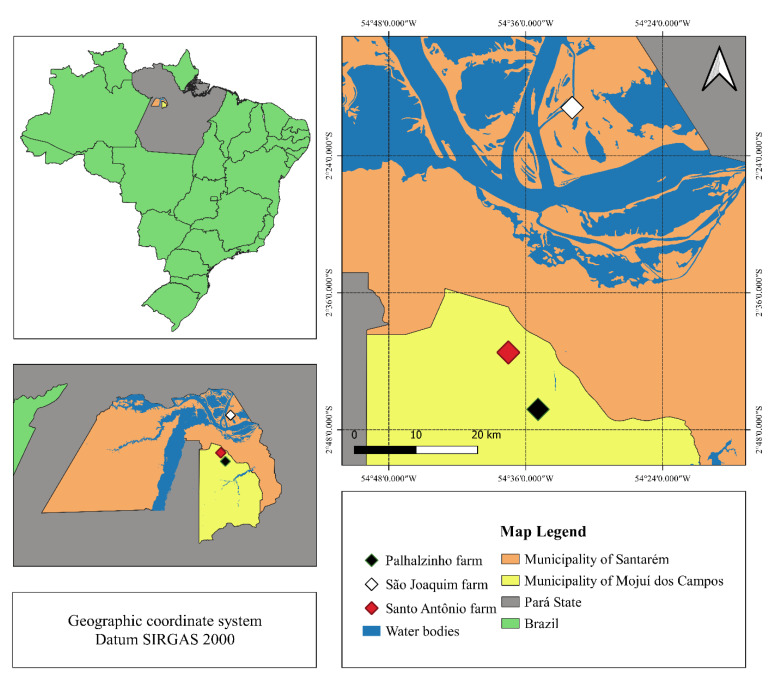
The location of the farms where ticks were collected.

The collection of ticks was carried out on commercial farms in the Lower Amazon region (Figure 1), which refers to the northwest quarter of the state of Pará and includes 13 municipalities, most of which are located along the Amazon River [17]. In this region, the cattle production system is based on a transportation pattern between the dry lands and the wetlands (*várzeas*) to benefit from the natural plains with grass on the margin of the Amazon River that became available with the drought [9]. In this system, cattle and buffalo from different farms were mixed yearly during the wetland season in areas without fences or any additional management of the herds. For this study, ticks were sampled on farms on dry lands after the return of cattle from wetlands. The herds were examined, and the engorged females were manually removed. The infested animals remained for 60 days without any acaricide treatment before collection, as they had just returned from the wetlands. The ticks were stored in plastic tubes with a perforated lid for air circulation and transported to the laboratory for resistance testing.

### 2.2. The Adult Immersion Test

The adult immersion test was performed according to the methodology described by [18]. The tests were carried out seven hours after the collection of ticks. For this, 150 engorged females were selected and washed in running water and dried with paper towels. Ticks were identified using a morphological key and distributed in 15 Petri dishes with 10 individuals each and homogenized by size. The Petri dishes were weighed on an analytical balance to determine the mass of the females and then distributed according to the treatments: 3 plates (*n* = 30) were used as a negative control (distilled water), and the remaining 12 plates were for the 3 groups with the acaricidal drugs (4 plates for each drug, *n* = 40). The three products used in the treatment were Amitraz (Ibatox, IBASA, Porto Alegre, Brazil), Cypermethrin (Barrage^®^, Zoetis Brazil, Campinas, Brazil), and Deltamethrin (Butox^®^ P CE 25, MDS Animal Health Brazil, São Paulo, Brazil).

Acaricides were prepared according to the manufacturers’ recommendations in solutions with a final volume of 250 mL for each product, using distilled water as a diluent. Amitraz solution was prepared with a concentration of 0.125 g/mL, Cypermethrin with a concentration of 0.15 g/mL, and Deltamethrin with a concentration of 0.025 g/mL. The diluted solutions were homogenized after preparation and immediately before testing. Distilled water was used for the control group.

The engorged ticks were submerged in the treatment and control solutions for five minutes, then briefly dried on paper towels and returned to the Petri dishes. The plates were placed in a B.O.D (Biological Oxygen Demand) oven with a temperature of 28 °C (±1 °C) and a relative humidity ≥80%. After 24 h of the acaricidal exposure, the ticks were examined with a stereomicroscope to check the mortality, with the counting of live and dead tick.

### 2.3. Evaluation of Acaricidal Efficiency

Acaricide efficiency was evaluated by the effect on the estimated reproduction index (ER) in the treatment groups. Therefore, the engorged females were kept in Petri dishes in a controlled environment until oviposition. After oviposition, the eggs were weighed and separated into plastic syringes sealed with hydrophilic cotton and kept in the same controlled conditions as the engorged females. Afterward, the hatchability of the eggs was visually analyzed according to the method described by [19,20]. To determine whether *R. microplus* is resistant to the treatment, we first calculate the estimated reproduction index (ER) with the formula: ER = egg mass/pre-oviposition female weight × hatching percentage × 20,000.

Then, the acaricidal efficacy was calculated according to FAO [21] using the mean values of the test repetitions of the control and treated groups, resulting in the acaricidal efficacy in vitro using the formula: acaricidal efficacy (AE) = (mean ER control group − mean ER treated group)/mean ER control group × 100.

The averages found were analyzed by Analysis of Variance (ANOVA), followed by Tukey’s test (*p* < 0.05). Statistical analyzes were performed using GraphPad Prism 9.4.0 software (GraphPad Inc., San Diego, CA, USA).

## 3. Results

The mortality of females treated with Cypermethrin was 7.5%, Amitraz 2.5%, and Deltamethrin and the negative control did not cause the death of the engorged females (Table 1).

**Figure 2:**
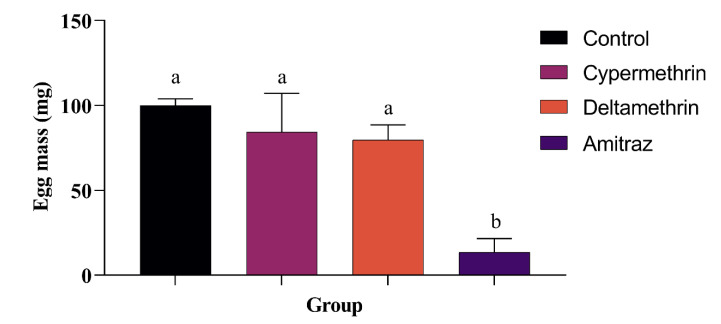
Comparison of average egg mass weight between groups. Columns with different letters differed significantly (*p* < 0.01).

The weight of egg mass produced by females treated with Amitraz was significantly lower than the mass of eggs produced by the other groups (Figure 2). While females exposed to Cypermethrin and Deltamethrin produced a mass of eggs with a weight statistically similar to the control group. However, there was no significant difference (*p* = 0.0534) in the percentage of larval hatching between groups (Table 2).

Table 2 presents the results obtained in the tick evaluation after the immersion in the acaricides. The group treated with Cypermethrin had the highest rate of egg production and the highest percentage of larval hatching. Deltamethrin had an egg production index of 39.6 and larval hatching of 80.0%. The group submerged in Amitraz had the lowest rates of egg production and hatching (7.1 and 53.7%, respectively), and the control group had an approximate hatching percentage of 99%.

**Figure 3:**
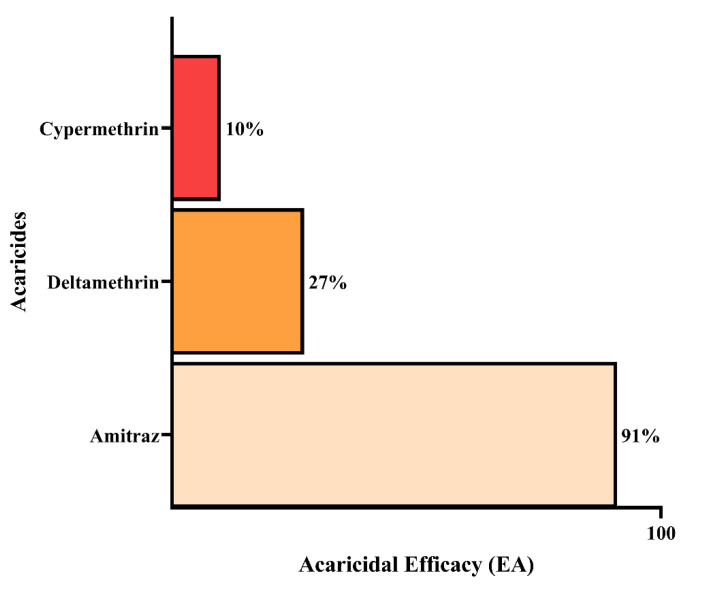
Acaricidal efficacy of Amitraz, Cypermethrin, and Deltamethrin against *R.microplus* engorged females.

Cypermethrin had the lowest rate of effectiveness, with only 10.4% control. Despite being the acaricide that caused the highest mortality of adult ticks, it did not show the ability to reduce the production of eggs and larvae, that is, it was not able to interfere with the cycle of *R. microplus*. In addition to not causing the death of any tick, Deltamethrin was also unable to effectively control the production of eggs and larvae with an efficacy rate of 26.6%. Amitraz was the most effective product, with 90.5% control (Figure 3). However, according to the criteria established by FAO [21], it is not considered effective because it has not reached the 95% control criterion.

## 4. Discussion

Under the conditions of our study, using tick populations from the Lower Amazon, Amitraz was not considered effective since it did not show an efficiency above 95% as recommended by FAO [21], as the results found in Paraná, where Amitraz (0.025%) was 97.4% to 100% effective [22]. However, Amitraz (0.025%) had an effectiveness of 90% in our study, higher than other regions, such as 84.6% in the Northeast, where the authors used a much lower dose of the acaricide (0.025%) [23] and in the Mato Grosso do Sul with 64.7% in properties with intense use [24].

The alternations in the effectiveness of Amitraz in different places may be related to the history of this control in these regions [25]. Amitraz inhibits egg laying and larval hatching due to its octopamine action [26]. However, the more the product is used, the more individuals end up generating mutations that can be passed on to their progeny, so acaricides with a long period of use have greater resistance compared to those with recent use [27].

In our study, Cypermethrin was the compound with the lowest efficiency compared to other products. Similar results were also reported in Mato Grosso do Sul with an average effectiveness of 19.9% [24], and in Canoinhas (SC), where a study with different dosages presented mean efficacy of Cypermethrin of 25.7%, 8.6%, and 3.5% [28]. In a study carried out in two rural properties in Rolim de Moura, RO, in vitro tests showed 81.3% of control using Cypermethrin on one farm and 23.9% on the other [16].

This acaricide, like other pyrethroid bases, acts on the sodium channels of the parasite and has a function in the nerve impulse, causing hyperexcitation and death [29]. The emergence of resistance can be explained by alterations of the acaricide in penetrating the individual [30]. This can be generated as a result of the time of exposure to the product since it takes two years for *R. microplus* to acquire resistance to pyrethroids under conditions of selection pressure [31].

Deltamethrin showed an efficacy of 26.6%, much lower than the 95% recommended by FAO. Studies have also reported low efficacy in the regions of Bahia, 65.0% [32], and Paraná, 42.3% [33]. Due to many reports of cattle tick resistance to pyrethroid compounds in Brazil, it should be used with more caution and only in the absence of resistance [34].

Acaricides can act on the parasite in three ways: direct ixodicidal action when the number of ticks decreases, including the entire development phase; ovariostatic action, which is the decrease in egg production; and by anti-embryogenic action, where the larvae’s hatching capacity decreases [35]. *R. microplus* has great reproductive potential that, together with the intense use of these acaricides, ends up selecting resistant populations [36]. This changes the acaricide’s capacity to control the parasite, which decreases its effectiveness.

Detecting resistance to these products is critical to improving management, delaying their development, and ensuring the sustainable use of the acaricide [37], demonstrating that it is necessary to make rational use of these products to maintain good efficacy and to adopt new control methods, such as those associated with chemical control [38], such as synthetic and botanical acaricides, and the use of vaccines [39].

## 5. Conclusions

This is the first study to evaluate resistance to commercial acaricides carried out in the western region of Pará. All the acaricide chemical bases evaluated, Amitraz, Cypermethrin, and Deltamethrin, showed efficacy below 95% against *R. microplus*, allowing the conclusion that there are strains resistant to commercial acaricides used in this region.

## Figures and Tables

**Table 1:** Mortality of engorged female *R. microplus* ticks after immersion in different acaricides.

Treatment	Average Mortality (%)
Amitraz	2.5
Cypermethrin	7.5
Deltamethrin	0
Negative control	0

**Table 2:** Reproductive parameters of *R. microplus* ticks after the adult immersion test with different acaricidal acaricide products.

Treatments	Mean Weight of Engorged Ticks before Oviposition (mg)	Egg Mass Weight (mg)	Egg Production Index (EPI) *	Percentage of Larval Hatching (%)
Amitraz	176.5	13.3	7.1	53.7
Cypermethrin	195.2	84.4	42.7	88.7
Deltamethrin	202.9	79.7	39.6	80.0
Negative control	184.2	80.2	42.3	98.3

* The proportion of female weight transformed into egg mass calculated by dividing the tick’s initial weight by the weight of the egg mass.

## Data Availability

The raw data of this work is available upon request to the corresponding author.
